# Adiponectin, Leptin, and Chemerin in Elderly Patients with Type 2 Diabetes Mellitus: A Close Linkage with Obesity and Length of the Disease

**DOI:** 10.1155/2014/701915

**Published:** 2014-07-02

**Authors:** Susana Coimbra, Jorge Brandão Proença, Alice Santos-Silva, Maria João Neuparth

**Affiliations:** ^1^Instituto de Biologia Molecular e Celular (IBMC), Universidade do Porto, Rua do Campo Alegre 823, 4150 Porto, Portugal; ^2^CESPU, Instituto de Investigação e Formação Avançada em Ciências e Tecnologias da Saúde (IINFACTS), Rua Central da Gandra 1317, 4585-116 Gandra, Portugal; ^3^Laboratório de Bioquímica, Departamento de Ciências Biológicas, Faculdade de Farmácia (FFUP), Universidade do Porto, Rua de Jorge Viterbo Ferreira 228, 4050-313 Porto, Portugal; ^4^Centro de Investigação em Actividade Física, Saúde e Lazer (CIAFEL), Universidade do Porto, Rua Dr. Plácido Costa 91, 4200-450 Porto, Portugal

## Abstract

Obesity, insulin resistance, and aging are closely associated and adipokines seem to have a crucial role in their pathophysiology. We aim to study the relationship between aging and chemerin, adiponectin, and leptin levels in type 2 diabetes mellitus (T2DM). Age correlated positively with chemerin and leptin and inversely with adiponectin. Body mass index (BMI) correlated positively with leptin (in males) and chemerin and inversely with adiponectin. The patients with ≥65 years (*n* = 34) showed significantly higher leptin and chemerin and lower adiponectin levels than middle-aged (38–64 years) patients (*n* = 39) and controls (*n* = 20). After statistical adjustment for length of disease, there was a loss of significance, between T2DM groups, for adiponectin and, in female, for leptin. In the older group, BMI correlated with adiponectin and with leptin, but not with chemerin. Adiponectin and leptin levels in elderly T2DM patients seem to be closely linked to obesity and to length of the disease. In elderly T2DM patients, chemerin concentrations are increased and seem to be independent of length of disease and BMI, suggesting that adipocyte dysfunction is enhanced with aging. The understanding of the glucose homeostasis impairment in the elderly is mandatory in order to achieve ways to improve their quality of life and longevity.

## 1. Introduction

The world population is rapidly aging and, along with this, the prevalence of insulin resistance and diabetes is increasing; both conditions are more prevalent in elderly humans [1]. Type 2 diabetes mellitus (T2DM) is more common at middle age and afterwards [2]. The impairment of glucose metabolism in older adults has been associated with a complex group of risk factors that contribute to increase insulin resistance with aging. Increasing urbanization, aging of the populations, obesity, and falling levels of physical activity are important contributors to the rise of diabetes mellitus worldwide [3].

The growing prevalence of obesity has been reported as the leading cause of DM prevalence [3]. Indeed, obesity, especially abdominal obesity, is a strong risk factor for cardiovascular disease (CVD) events and for insulin resistance that, usually, leads to T2DM [4, 5]. A hallmark of human aging is an increase of adipose mass, especially of visceral adipose mass, which is an independent risk factor for chronic heart failure in older people [6]. The age-related increase in hypertension, dyslipidemia, and impaired glucose metabolism, the known cluster factors for the metabolic syndrome, seems to be associated with the increasing prevalence of overweight and obesity in the elderly. Thus, obesity, insulin resistance, and aging appear to be closely linked.

Adipose tissue is an active endocrine organ that secretes several inflammatory cytokines, namely, adipokines, which interfere with insulin sensitivity, with glucose and lipid metabolism, and with the inflammatory process [3, 7]. The pathophysiologic link between obesity and T2DM is not entirely understood, but adipokines seem to play an important role.

Adiponectin is an adipose tissue specific cytokine that has a protective role against insulin resistance [8] and anti-inflammatory activity and seems to protect against metabolic diseases [9]. There are different circulating forms of adiponectin complexes, low molecular weight (LMW), medium molecular weight (MMW), and high molecular weight (HMW) adiponectin; the increase of HMW adiponectin has been reported as an even better biomarker of metabolic stress than total adiponectin. Leptin, another adipokine, enhances the secretion of several cytokines by inflammatory cells and a reduction in its activity leads to insulin resistance [10, 11]. Both adiponectin and leptin levels are known to be altered in obesity [12–15]. According to a recent study of our group, adiponectin and leptin levels in T2DM patients are more associated with obesity and less with diabetes [16]. Actually, high adiponectin levels were associated with a lower incidence of T2DM [17]. Chemerin, a more recently identified adipose tissue specific adipokine, has a crucial role in adipocyte differentiation and development, as well as in glucose and lipid metabolism [18, 19]. Raised chemerin levels were found in obese subjects, in prediabetic states, and in lean, overweight, and obese T2DM patients [16, 20, 21].

A study on octogenarians with T2DM showed that total adiponectin, HMW, and MMW adiponectin presented significantly higher serum levels, when compared with middle-aged patients with T2DM [22]. As far as we know, there are no studies concerning leptin and chemerin levels in older patients with T2DM.

Our aim was to study the relationship between aging and the levels of the adipokines, chemerin, adiponectin, and leptin, in T2DM patients, by studying middle-aged patients (38–64 years old) and old patients, with more than 65 years old.

## 2. Material and Methods

### 2.1. Subjects

The protocol used was approved by the Committee on Ethics of the Instituto Superior das Ciências da Saúde Norte (CESPU), Gandra, Portugal.

We performed a cross-sectional study on Portuguese adult patients with T2DM. A group of 73 patients with T2DM selected from the general population was enrolled in this study, after their informed consent. Patients presenting inflammatory or infectious diseases and liver or kidney diseases and receiving any kind of medication that could interfere with the study evaluations were not included in the study. An accurate and detailed interview with the patients was performed in order to collect and record the clinical characteristics of the disease, sociodemographic data, and their habits. Patients whose statements were confusing, unclear, and inconsistent were excluded from the study. Patients had been diagnosed for a long time with T2DM; the length of the disease was 9 ± 7 years old (mean ± standard deviation (SD)). T2DM patients reported that their diet was low in carbohydrates and fat; all were under treatment with oral hypoglycemic drugs (sulfonylureas or biguanides).

Patients were divided into 2 groups, according to age, the middle-aged group, which included patients of 38–64 years old (56 ± 7 years old; *n* = 39), and the older-aged group with 65–85-year-old patients (73 ± 5 years old; *n* = 34). Both groups of patients were matched for gender (19/18 females and 20/16 males, in the middle and in the older groups, resp.). The length of disease was 8 ± 6 years for the younger group and 11 ± 8 years for the oldest group; the difference between the groups was almost not significant (*P* = 0.047).

The control group included 20 volunteers without previous diagnosis of T2DM, who were not under any medication and matched with both groups of T2DM patients, for gender, body mass index (BMI), smoking (20% smokers, 16 cigarettes/day for controls; 18% smokers, 17 cigarettes/day for middle-aged group; 15% smokers, 19 cigarettes/day for older-aged group), and alcohol drinking habits (55% consume alcohol, 10 wine glasses/week for controls; 59% consume alcohol, 10 wine glasses/week for middle-aged group; 53% consume alcohol, 10 wine glasses/week for older-aged group); only the middle-aged group of patients was matched for age with the control.

The type of medication, nutrition intake, leisure activities, smoking, and alcohol drinking habits were similar in both groups of T2DM patients. Besides the oral hypoglycemic therapy, none of the patients were receiving any medication that could interfere with our results (e.g., antioxidants, anti-inflammatory drugs, and antiobesity therapies).

### 2.2. Assays

Blood from fasted (12 hours) subjects was collected into tubes without anticoagulant in order to obtain serum. None of the collected samples was icteric or hemolysed.

Adipokines were evaluated by enzyme immunoassays (Human Adiponectin, R&D Systems, Minneapolis, USA; Leptin ELISA, Mercodia, Uppsala, Sweden; Human Chemerin ELISA, Biovendor Research and Diagnostic Products, Heidelberg, Germany). The lipid profile (cholesterol, triglycerides, and high-density lipoprotein cholesterol (HDLc)) and glucose were evaluated by enzymatic colorimetric methods (Prestige, PZ Cormay, Lublin, Poland). To determine the levels of glycated hemoglobin, we used a spectrophotometric method (Prestige 24i HbA_1C_, PZ Cormay, Lublin, Poland).

### 2.3. Statistical Analysis

We used the Statistical Package for Social Sciences (SPSS, version 17 for Windows, Chicago, IL, USA). A *P* value lower than 0.05 was considered as statistically significant. Comparisons between groups were performed using Student's unpaired *t*-test or Mann-Whitney *U* test, according to Gaussian distribution of the substances. Measurements are expressed as mean ± SD or as median values (interquartile range), in accordance with Gaussian distribution. Adjustment for confounding factors was performed using analysis of covariance (ANCOVA), after transformation of variables (when necessary); variables were linearized by logarithmic transformation and, afterwards, checked for a Gaussian distribution. The correlation analysis was performed by calculating the Spearman coefficient correlation. The multiple regression analysis was performed using stepwise selection with an entry criteria of *P* < 0.05. All variables included in the regression analysis respected a linear distribution; when necessary, variables were linearized and checked for normality.

## 3. Results

Sociodemographic, clinical, and analytical data of the control group and T2DM patients are presented in Table 1.

As referred, T2DM patients and controls were matched for gender, age, and BMI. Patients with T2DM presented significantly lower levels of adiponectin and higher leptin and chemerin values, as compared to controls. Concerning subject gender, we found significantly higher leptin values for female controls and patients; in addition, female T2DM patients presented significantly higher values of leptin, as compared to female controls (Table 1). The female control group (*n* = 12), that included subjects with 62 ± 8 years old and BMI of 27 ± 2 kg/m^2^, was matched with female T2DM patients (*n* = 37), who were 64 ± 11 years old (*P* = 0.508) and with a BMI of 28 ± 3 kg/m^2^ (*P* = 0.276). The male control group (*n* = 8) included subjects of 57 ± 10 years old with a BMI of 25 ± 3 kg/m^2^ and was matched with male T2DM patients (*n* = 36) which included patients of 64 ± 9 years old (*P* = 0.075) with a BMI of 26 ± 4 kg/m^2^ (*P* = 0.634).

**Figure 1:**
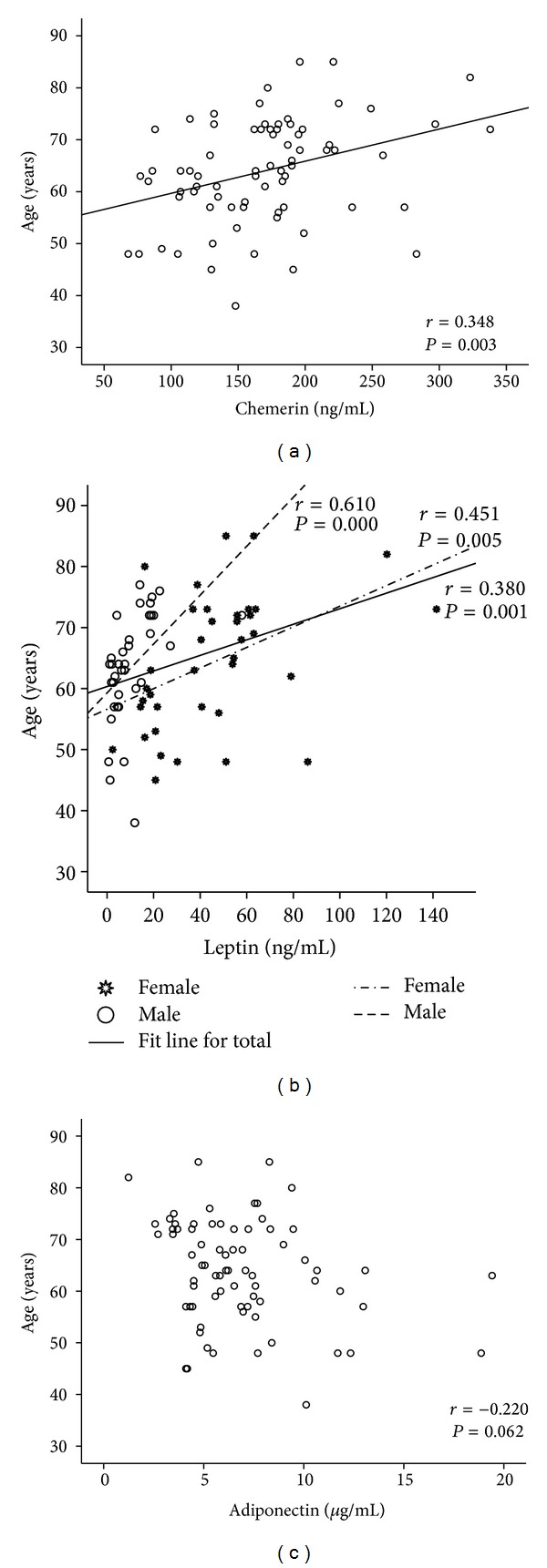
Correlations found between age and levels of chemerin (a), leptin (b), and adiponectin (c).

When considering all T2DM patients, their age correlated significantly and positively with chemerin (Figure 1(a)) and leptin levels (Figure 1(b)); a trend towards an inverse correlation with adiponectin (Figure 1(c)) values was also observed. Concerning leptin, this correlation was observed in both females and males (Figure 1(b)). By performing multiple linear regression analysis, considering adiponectin, leptin, and chemerin, we found that only Lg_10_ leptin was significantly associated with the age of T2DM patients (*β* = 0.371; *P* = 0.001). Moreover, in T2DM patients, BMI was significantly and positively correlated with chemerin (*r* = 0.407, *P* < 0.001) and leptin (*r* = 0.490, *P* < 0.001; males: *r* = 0.450, *P* = 0.005; females: *r* = 0.277, *P* = 0.102) and inversely correlated with adiponectin (*r* = −0.419, *P* < 0.001). Multiple linear regression analysis showed that Lg_10_ leptin and Lg_10_ adiponectin were significantly associated with the BMI of T2DM patients (*β* = 0.352, *P* = 0.003 and *β* = −0.266, *P* = 0.025, resp.).

Analysing our results in accordance with age groups, we found that glucose, glycated hemoglobin, total cholesterol, and HDL cholesterol presented similar values for both groups. The older-aged group, as compared to the middle-aged group, showed significantly higher levels of leptin (in both genders) and chemerin and lower values of adiponectin (Table 2). After statistical adjustment for length of disease, there was a loss of significance for adiponectin (*P* = 0.119) and for leptin in female patients (*P* = 0.117). The female middle-aged group (*n* = 19) included subjects of 55 ± 6 years old with a BMI of 28 ± 3 kg/m^2^ and the male middle-aged patients (*n* = 20) were 58 ± 7 years old with a BMI of 25 ± 4 kg/m^2^. The female older-aged subjects (*n* = 18) were 74 ± 6 years old with a BMI of 28 ± 4 kg/m^2^ and the male patients (*n* = 16) were 71 ± 4 years old with a BMI of 27 ± 4 kg/m^2^. When comparing middle-aged and older-aged groups, female (*P* = 0.469) and male (*P* = 0.205) were matched for BMI.

The middle-aged group, as compared to controls, presented significantly lower levels of adiponectin (*P* = 0.019)and higher levels of chemerin (*P* < 0.001). The older-aged group, as compared to controls, showed significantly higher levels of chemerin (*P* < 0.001) and leptin (*P* < 0.001), both in female (*P* < 0.001) and in male patients (*P* = 0.002) and lower values of adiponectin (*P* < 0.001). As previously referred, the control group was matched for gender and BMI with T2DM patients; however, considering the age groups, only the middle-aged group was matched for age with the control group. Therefore, we performed an adjustment for age, and we found that all differences remained statistically significant for the older group, except leptin values for males that lost statistical significance (*P* = 0.069).

In the middle-aged group, BMI correlated with adiponectin (*r* = −0.345; *P* = 0.032), leptin (*r* = 0.485; *P* = 0.002) at least in females (*r* = 0.517; *P* = 0.024), and chemerin (*r* = 0.527; *P* = 0.001). In the older group, BMI correlated with adiponectin (*r* = −0.475; *P* = 0.005) and with leptin when considering both genders together (*r* = 0.423; *P* = 0.013), but not with chemerin (*r* = 0.190; *P* = 0.282).

## 4. Discussion

Adiposity and altered adipokine secretion seem to predispose to the development of type 2 diabetes. Adiponectin plays a protective role against insulin resistance [8] and has anti-inflammatory activity [9], protecting also against atherosclerosis [23]. However, the protective role against cardiovascular diseases is controversial, with several data reporting a positive association of adiponectin with risk for all-cause and cardiovascular mortality [24–26]. It has been proposed that, paradoxically, higher circulating levels of adiponectin might be a signal of lower diabetes risk and, simultaneously, a signal of higher CVD risk.

Leptin leads to increased levels of several proinflammatory cytokines and the lack of its activity leads to insulin resistance [10, 11]. The development of resistance to leptin effects has been proposed to explain its reduced activity in obesity [27].

Chemerin plays a vital role in adipocyte differentiation and development, and it may act as a modulator of different metabolic pathways in mature adipocyte [18, 19], namely, in the expression of adipocyte genes involved in glucose and lipid homeostasis [19]. Moreover, chemerin associates with several metabolic syndrome markers, such as BMI, triglycerides, blood pressure, and insulin resistance [28, 29]. It was reported recently that insulin resistance seems to be a predictor of chemerin levels, independent of BMI [30].

In the present work, the studied Portuguese patients with T2DM, as compared to controls, presented lower levels of adiponectin and higher values of chemerin and leptin, in accordance with published data [13, 21, 31].

As referred, aging is known to be associated with increasing insulin resistance, weight, and adiposity gain.    According to our data, aging in T2DM patients is associated with an altered adipokine secretion, as shown by the significant and positive correlations with leptin and chemerin and the inverse correlation with adiponectin. To further analyze these results in accordance with age, we studied and compared middle-aged and older T2DM patients.

Concerning adiponectin, controversial data exists in literature; some authors reported that, in healthy subjects, its levels increase with age [32], while others found those changes only for males [33]; in opposition, Vilarrasa et al. referred to a decline in adipokine levels with age [34]. A decrease in adiponectin clearance by the kidney was pointed as a possible cause for the adiponectin increase with age [32], while others suggested that the high levels of adiponectin in those with acute coronary syndrome or heart failure may be the reflection of a salvage mechanism to improve insulin resistance and fatty acid metabolism [35]. Higher adiponectin levels were strongly associated with a lower risk for impaired glucose metabolism and T2DM, suggesting that adiponectin is involved in the pathophysiologic mechanisms linking obesity to type 2 diabetes [17]. Indeed, treatment of elderly T2DM patients with glimepiride improved insulin resistance, which was linked to an increase in adiponectin and a decrease in tumour necrosis factor-*α* levels [36]. According to Wannamethee et al., the association between low adiponectin and an increased risk for diabetes appears to be significantly stronger in older obese men than in leaner counterparts; low adiponectin levels were also associated with increased risk of T2DM even after adjustment for BMI, lifestyle factors, preexisting cardiovascular disease, and systolic blood pressure [37]. As referred, a study of octogenarian patients with T2DM showed that total adiponectin, as well as HMW and MMW adiponectin, levels were significantly higher, as compared with middle-aged patients with T2DM [22].

In our study, we found that the older T2DM patients presented significantly lower adiponectin levels, when compared with middle-aged T2DM patients and with controls. However, after statistical adjustment for length of disease, the difference in adiponectin levels found between age groups was lost. The difference between our results and those from Graessler et al. [22] might be related to the age of the patients and/or to the adipose mass content, since our patients were younger (73 ± 5 years old) and presented a trend towards higher BMI. We must emphasize that we found a significant inverse association between adiponectin and BMI values, as already observed by others [34], when considering all T2DM patients and when considering middle-aged or older patients separately, suggesting that, in T2DM patients, adiponectin levels are dependent on BMI values. Besides, as the significant difference of adiponectin levels found between T2DM age groups was lost after adjustment for length of the disease, it seems that, in T2DM patients, adiponectin levels are also dependent on the length of the disease.

Data concerning the relationship between leptin levels and aging in healthy population is also controversial. While some authors reported that leptin decreases with age [38], others did not find any change [39–41]; and others referred to an increase and a positive correlation of leptin with aging in elderly subjects [42, 43], at least in the overweight elderly [44]. Controversial data also exists concerning the relationship of leptin with BMI; some data refer to a positive association between leptin and BMI in the elderly [42, 43, 45], while others reported that leptin values are independent of BMI [38]. Aging has been also associated with resistance to leptin and/or with a decrease in leptin receptors [44]. The study by Wannamethee et al. in older men (60–79 years old) showed that the association between leptin and incident diabetes was mediated by insulin resistance and that high leptin levels were associated with increased risk for T2DM, even after adjustment for BMI, lifestyle factors, preexisting cardiovascular disease, and systolic blood pressure [37]. Another study in elderly men showed that leptin was increased and positively associated with all metabolic syndrome components, namely, with insulin resistance and abdominal fat [46]; aging was associated with an increase in insulin resistance and with a decrease in glucose uptake, which is influenced by leptin. Our data in elderly Portuguese T2DM patients are in accordance with these studies showing an increase in leptin levels; however, after statistical adjustment for length of the disease, the significant increase in leptin for older females, as compared to middle-aged females, was lost, suggesting that leptin levels are also dependent on the length of T2DM. Moreover, we found that leptin values in T2DM patients were positively associated with BMI. Increasing leptin might be regarded as a marker of risk as it has been associated with insulin resistance, increased secretion of proinflammatory cytokines, increased onset of depressive symptoms [47], T2DM, and estimated age-related cognitive change in elderly men [48].

Data regarding the relationship of chemerin levels with age are scarce; but, according to Aronis et al., age is positively correlated with chemerin [49]. As far as we know, there is no evaluation of the relationship between chemerin levels and age in T2DM. Our results showed that older T2DM patients presented higher chemerin levels compared to middle-aged T2DM group and to controls; these results strengthen the proposal that chemerin increases insulin resistance, which increases with age. After statistical adjustment for length of disease, the significant increase of chemerin levels in the oldest T2DM group remained significant, suggesting that chemerin levels are dependent on age. In spite of the correlation observed between chemerin and BMI when considering all T2DM patients, we found that when considering the older group of patients this correlation was no longer observed, suggesting that, in older T2DM patients, the chemerin values are not dependent on BMI. Indeed, insulin resistance seems to be a predictor of chemerin levels, independent of BMI [30]; actually, in a recent study from our group, we found that chemerin was raised in lean, overweight, and obese T2DM patients [16]. It seems that in T2DM, independently of BMI, a dysfunction in adipose tissue occurs and is enhanced with age.

A comprehensive understanding of the impairment in glucose homeostasis in the elderly is necessary in order to achieve ways to improve their quality of life and longevity. Older T2DM patients, by presenting an altered adipokine secretion, may be at a higher risk for CVD events. These patients should be encouraged to adopt strategies to reduce the CVD risk, by implementing healthy diet habits and physical exercise practice. Indeed, we reported recently that the practice of moderate walking on a regular basis was sufficient to reduce chemerin levels in T2DM patients [50], which may improve their insulin sensitivity and lipid profile.

## 5. Conclusions

In summary, adiponectin and leptin levels in elderly patients with T2DM seem to be closely linked to obesity and to length of the disease. In the older group of T2DM patients, circulating chemerin concentrations are increased and seem to be independent of the length of disease and BMI, suggesting that adipocyte dysfunction is enhanced with aging.

## Figures and Tables

**Table 1:** Sociodemographic, clinical, and analytical data for control group and patients with type 2 diabetes mellitus (T2DM).

	Control group (*n* = 20)	T2DM patients (*n* = 73)	*P* value
Gender (F/M)	12/8	37/36	0.399
Age (years)	60 ± 9	64 ± 10	0.127
Length of disease (years)	—	9 ± 7	—
Body mass index (kg/m^2^)	26 ± 3	27 ± 4	0.304
Total cholesterol (mg/dL)	210 ± 48	196 ± 52	0.276
HDL cholesterol (mg/dL)	46 ± 18	40 ± 10	0.173
Triglycerides (mg/dL)	97 [64–184]	107 [78–155]	0.633
Adiponectin (*μ*g/mL)	8.3 [6.8–14.2]	6.1 [4.5–7.9]	0.001
Leptin (ng/mL)	6.7 [4.6–14.7]	18.6 [7.1–46.6]	0.004
Male	4.9 [2.2–11.0]^a^	7.4 [3.2–17.3]^b^	0.216
Female	15.3 [6.7–25.2]	43.0 [20.8–59.3]	0.002
Chemerin (ng/mL)	89 [66–109]	179 [130–193]	<0.001

F: female; HDL: high-density lipoprotein; M: male.

^a^ *P*: male versus female <0.01; ^b^ *P*: male versus female <0.001.

**Table 2:** Body mass index, biochemical, and adipokine data according to age of T2DM patients.

	T2DM middle-aged group (*n* = 39)	T2DM older group (*n* = 34)	*P* value
Gender (F/M)	19/20	18/16	0.721
Age (years)	56 ± 7	73 ± 5	<0.001
Length of disease (years)	8 ± 6	11 ± 8	0.047
Body mass index (kg/m^2^)	26 ± 4	28 ± 4	0.135
Glucose (mg/dL)	130 [106–172]	126 [100–162]	0.812
Glycated hemoglobin (%)	6.7 [6.1–8.0]	7.1 [6.5–8.0]	0.803
Total cholesterol (mg/dL)	195 ± 56	197 ± 47	0.876
HDL cholesterol (mg/dL)	40 ± 10	40 ± 11	0.970
Triglycerides (mg/dL)	99 [77–152]	129 [92–180]	0.157
Adiponectin (*μ*g/mL)	7.0 [5.2–10.1]	5.4 [3.7–7.6]	0.012∗
Leptin (ng/mL)	12.4 [3.4–21.6]	37.9 [17.7–57.8]	<0.001
Male	4.8 [2.0–7.5]^a^	18.4 [9.4–19.8]^a^	<0.001
Female	21.6 [17.0–48.0]	55.9 [42.4–63.0]	0.001∗
Chemerin (ng/mL)	145 [107–180]	188 [169–219]	<0.001

F: female; HDL: high-density lipoprotein; M: male.

∗Loss of significance after statistical adjustment for length of disease.

^a^ *P*: male versus female <0.001.
